# The bidirectional relationship between homebound status and falls among older adults: a longitudinal study

**DOI:** 10.1186/s12877-022-03588-1

**Published:** 2022-12-01

**Authors:** Minhui Liu, Wenting Peng, Christina E. Miyawaki, Cen Mo, Yuqian Luo, Ni Gong

**Affiliations:** 1 Xiangya School of Nursing, Central South University, Changsha, China; 2 Graduate College of Social Work, University of Houston, Houston, TX USA; 3 The Third Xiangya Hospital of Central South University, 138 Tongzipo Road of Yuelu District, Changsha, 410013 China

**Keywords:** Bidirectional association, Community-dwelling, Falls, Homebound status, Older adults

## Abstract

**Background:**

Previous research has shown an association between homebound status and falls among older adults. However, this association was primarily drawn from cross-sectional studies. This study aimed to determine the bidirectional relationship between homebound status and falls among older adults in the community.

**Methods:**

We used data of the community-dwelling older adults from 2011 to 2015 of the National Health and Aging Trends Study, a nationally representative survey of Medicare Beneficiaries in the United States (Sample 1 [No falls at baseline]: *N* = 2,512; Sample 2 [Non-homebound at baseline]: *N* = 2,916). Homebound status was determined by the frequency, difficulty, and needing help for outdoor mobility. Falls were ascertained by asking participants whether they had a fall in the last year. Generalized estimation equation models were used to examine the bidirectional association between homebound status and falls longitudinally.

**Results:**

Participants with no falls at baseline (*n* = 2,512) were on average, 76.8 years old, non-Hispanic whites (70.1%), and female (57.1%). After adjusting for demographics and health-related variables, prior year homebound status significantly contributed to falls in the following year (Odds ratio [OR], 1.28, 95% CI: 1.09–1.51). Participants who were non-homebound at baseline (*n* = 2,916) were on average, 75.7 years old, non-Hispanic white (74.8%), and female (55.8%). Previous falls significantly predicted later homebound status (OR, 1.26, 95% CI: 1.10–1.45) in the full adjusted model.

**Conclusion:**

This is the first longitudinal study to determine the bidirectional association between homebound status and falls. Homebound status and falls form a vicious circle and mutually reinforce each other over time. Our findings suggest the importance of developing programs and community activities that reduce falls and improve homebound status among older adults.

## Introduction

The World Health Organization estimates that approximately 22% of the global population will be 60 years and older by 2050 [1]. Falls are a serious public health concern for older adults because of the high prevalence, mortality, and adverse outcomes [2]. The one-year prevalence of falls ranges from 26 to 34% among adults ages between 65 and 85 in the United States (U.S.) [2]. Falls may cause severe consequences, such as fear of falling, fractures, and physical disability [3], resulting in a huge medical burden (more than $50 billion) to families and society [2]. Thus, it is essential to identify factors associated with falls.

Being homebound is defined as rarely or never leaving home which may lead to falls [4, 5]. Homebound older adults are characterized by loss of independence, severe functional impairments, and social isolation [6, 7]. In the U.S., the number of homebound older adults increased from 1.6 million in 2019 to 4.2 million in 2020 [8]. Nearly 23% of homebound older adults have experienced at least one fall during the last month, and that is three times more frequent compared to that of non-homebound counterparts [4]. In addition, homebound status is associated with mobility disorder, depression, and dementia [9], all of which are recognized risk factors for falls [3, 10].

Falls are also a significant risk factor for homebound status [11]. Falls-related outcomes such as hip fractures and fear of falling can prevent older adults from going outside due to limited physical function and motivation [12, 13]. Therefore, the homebound status may be associated with falls bidirectionally, given that they have shared some common risk factors such as vision impairments [6, 14], physical disability [3, 4] and depressive symptoms [15, 16].

We searched Web of Science and PubMed for articles published from database inception until November 2021, with the terms “falls” AND “homebound” OR “housebound”. We found nearly 140 articles in total. But most studies have focused on determining the risk factors or implementing intervention programs for falls among the homebound population. Only three cross-sectional studies specifically investigated the association between falls and homebound status among older adults. Two studies (one including 564 older adults with diabetes [11]; the other including 5,930 community-dwelling older adults [5]) found the association between falls and homebound status, but the third study with 878 older adults who were rural residents, found no significant relationship between falls and homebound status after adjusting for the confounders [17]. One possible explanation may be the collinearity of disabilities and falls [3, 17]. Overall, these studies were limited to poor sample representativeness and assessed cross-sectionally and unidirectionally.

From a clinical practice standpoint, older adults with falls and homebound older adults have complex medical needs because they tend to have a high prevalence of physical disability and mental disorders [18, 19]. Public healthcare providers face great challenges in assisting them to reduce adverse health outcomes, especially when falls and homebound status coexist because they are especially vulnerable and may require long-term home care [20, 21]. If the longitudinal bidirectional relationship between falls and homebound status exists, it may provide opportunities for developing preventive public health strategies for early screening and managing risk factors for both falls and homebound status in older adults. Additionally, to our knowledge, no interventions have been developed for reducing falls and homebound concurrently. The bidirectional relationship may be helpful in determining the potential effects of fall interventions on older adults at risk of becoming homebound and vice versa. Therefore, we aimed to examine the longitudinal bidirectional relations between homebound status and falls using a nationally representative sample of community-dwelling older adults in the U.S. Our study had two objectives: to determine if prior year homebound status predicts falls in the following year and if prior year falls predict homebound status in the following year.

## Methods

**Fig. 1:**
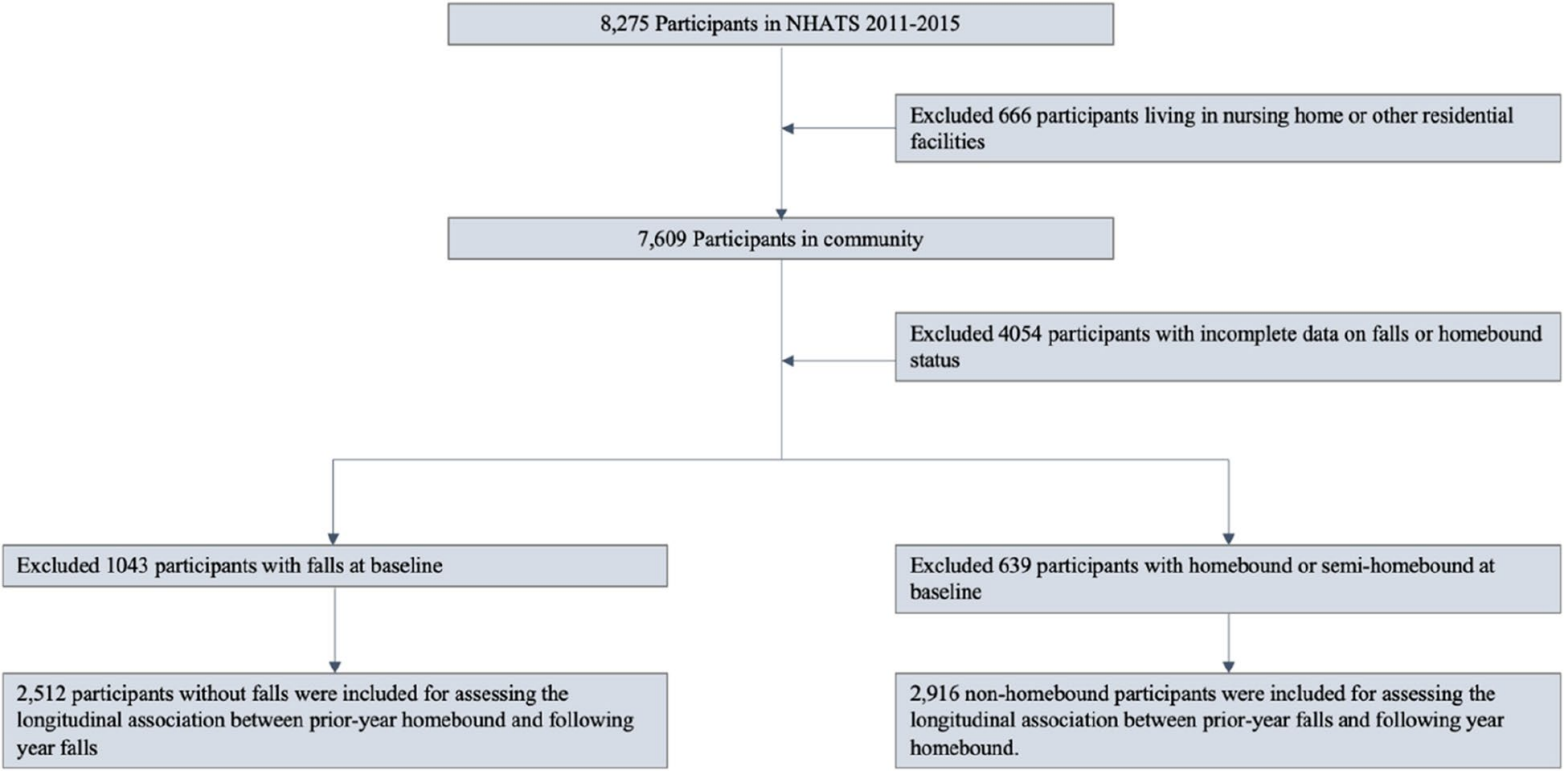
The flowchart of the selection of the two samples

We used data from 2011 to 2015 (Year 1 to Year 5) waves of the National Health and Aging Trends Study (NHATS), a nationally representative longitudinal cohort study of older adults in the U.S. who are Medicare beneficiaries aged 65 and older [22]. The NHATS was designed to understand the aging trend in late life. After excluding respondents with incomplete information on falls and homebound status, we created two samples: Sample 1 (*n* = 2,521) and 2 (*n* = 2,916). Sample 1 were community-dwelling older adults who reported no falls at baseline. Sample 2 were community-dwelling older adults who were non-homebound at baseline. Figure 1 presents the detailed selection procedure of study participants. The NHATS study used nonidentifiable data and was approved by the Johns Hopkins University Institutional Review Board. Participants completed written informed consent prior to being interviewed. The current analyses were deemed exempt from review by the Xiangya School of Nursing Ethic Committee of Central South University.

### Measurements

#### Falls

Falls were assessed by two questions: (a) “have you fallen down” and (b) “have you fallen down more than one time” over the last 12 months. The answers were yes or no. We classified falls as a binary variable (coded 0 for no falls and 1 for falls) in the main analysis, and a three-categorical variable (coded 0 = no falls; 1 = one fall; 2 = multiple falls) in the sensitivity analysis.

#### Homebound status

The prevalence of homebound status varied depending on different definitions. As suggested by Lee et al. [23], we measured the frequency of leaving the house and functional difficulty simultaneously and classified it as “semi-homebound” to differentiate it from homebound status. Based on measures developed by Ornstein and colleagues [4], in this study, homebound status was measured using four questions based on the reported frequency of outdoor mobility and functional difficulties. First, the participants were asked “How often did you go out in the last month?” and response options were on a 5-point Likert scale: never, rarely (≤ 1 day per week); some days (2–4 days per week); most days (5–6 days per week); and every day. Participants reporting going outside at least 2 days per week were asked whether they needed help from others to go outside. Those who reported needing help were also asked about the frequency of going outside independently. Those who reported never going outside by themselves were asked whether they had any difficulties leaving home independently.

Participants were classified as homebound if the frequency of going outside was less than or equal to 1 day per week. Participants were classified as semi-homebound if they could not go outside by themselves or they had any difficulties leaving the house by themselves. All other participants were classified as non-homebound. We classified homebound status as a binary variable (coded 0 for non-homebound and 1 for semi-homebound/homebound) in the main analysis and a three-categorical variable (coded 0 = non-homebound; 1 = semi-homebound; 2 = homebound) in the sensitivity analysis.

#### Covariates

We included the following demographic and health-related variables as covariates. We selected these variables based on similar previous studies, and they were commonly used variables and associated with falls and homebound status [7, 24–27].

Demographic variables included age, gender (0 = female; 1 = male), race (coded 0 to 3 for non-Hispanic white, non-Hispanic Black, Hispanic and other), education level (coded 0 to 3 for less than high school, high school graduates, college or vocational school, bachelor or higher), and living arrangement (coded 0 to 3 for alone, with spouse/partners only, with others only, with spouse/partner and others).

Health-related factors included following variables: Body Mass Index (BMI) (0 = normal; 1 = obesity[≥ 30 kg/m^2^]); bothersome pain was measured by asking participants whether bothered by pain in the last month (0 = no; 1 = yes); depressive symptoms were determined as a score of 3 or higher using the Patient Health Questionnaire-2 (0 = no; 1 = yes); dementia status was ascertained by a self-reported medical diagnosis of dementia or Alzheimer’s disease (0 = no; 1 = yes); whether the subjects were hospitalized in the last 12 months (0 = no; 1 = yes); number of activities of daily living (ADL) difficulties, including eating, dressing, toileting and bathing; number of instrumental activities of daily living (IADL) difficulties, including laundering, shopping, cooking, banking and taking medicines; number of chronic diseases (arthritis, cancer, diabetes, heart attack, heart disease, hypertension, lung disease, osteoporosis, and stroke); visual impairment was determined by asking participants whether they were blind or unable to see well enough to recognize people across the street or read newspaper print (0 = no; 1 = yes); hearing impairment was determined by asking participants whether they were deaf, wore the hearing aid or were unable to hear well enough to use the telephone or carry on a conversation while watching the television or listening to the radio (0 = no; 1 = yes); and whether they performed vigorous activities last month (0 = no; 1 = yes).

### Statistical analysis

Demographic and health-related characteristics were described using frequencies, proportions, means, and standard deviations (SDs). For categorical and continuous variables, we used Chi-square test and two-sample t-test to test the differences between participants stratified by homebound status in sample 1 and falls in sample 2. We used generalized estimation equation (GEE) models to test the lagged bidirectional association between falls and homebound status. The lagged GEE model incorporated within-subject correlation across repeated measurements and was useful to estimate the population-averaged effects over time [28]. The lagged GEE model was used to estimate whether the independent variable at time t was related to the outcome variable at time *t* + *1* after adjusting for the outcome variable at time *t.*

#### Homebound status as a predictor of falls

In sample 1, we performed a set of lagged GEE models with a binomial distribution, logit link function, and exchangeable within-group correlation structure [29]. Robust standard errors were used to account for correlation between measures for each measure [29]. We analyzed the lagged GEE models, in which homebound status at time t was related to falls at time *t* + *1* after adjusting for the falls at time *t*.

#### Falls as a predictor of semi-homebound or homebound

In sample 2, we performed a set of lagged GEE models, with a similar modeling strategy to the aforementioned models. We analyzed the lagged GEE models, in which falls at time t were related to semi-homebound or homebound at time *t* + *1* after adjusting for the semi-homebound or homebound at time *t*.

We presented three models for each lagged association. Model 1 included risk factors of interest only. Model 2 adjusted for demographics (age, gender, education, race, and living arrangement), and Model 3 further adjusted for health-related variables (BMI, pain, depressive symptoms, dementia, hospitalization, number of ADL/IADL impairments and chronic illness, hearing and vision impairment, vigorous activity).

Further, we performed two sensitivity analyses. First, in sample 1, we recoded homebound status as a three-categorical variable (coded 0 = non-homebound; 1 = semi-homebound; 2 = homebound) to predict future falls. Second, in sample 2, we recoded falls as a three-categorical variable (coded 0 = no falls; 1 = one fall; 2 = multiple falls) to predict future semi-homebound/homebound status.

Odds ratio (ORs) and 95% confidence intervals (CI) were reported. There were missing values on covariates less than 2.2% (BMI) in sample 1 and 2.0% (BMI) in sample 2. Given the large sample size, no particular technique was used to handle missing data. *P* value less than 0.05 indicated statistical significance. All analyses were performed in Stata SE version 15.0.

## Results

### Semi-homebound or homebound status as a predictor of falls

A total of 2,512 participants who reported no falls at baseline were included in sample 1, of which 14% were semi-homebound and homebound. Characteristics of all participants in sample 1 and their comparisons by homebound status were presented in Table 1 (*N* = 2,521). In sample 1, the mean age of all participants was 76.8 years old. Fifty-seven percent were female and 70% were non-Hispanic white. Compared to non-homebound participants, semi-homebound or homebound participants were more likely to be older female and people of color. They tended to be less educated, lived with others, experienced more pain, depressive symptoms, as well as dementia, reported more hospitalizations, higher numbers of ADL/IADL impairments and chronic diseases, vision and hearing problems, and performed less vigorous activities (*P* < 0.001 for all comparisons).

**Table 1:** Baseline characteristics of participants by homebound status (Sample 1, *N* = 2,521)

Characteristics	Sample 1			*P* value
	Total	Semi-homebound and homebound	Non-homebound	
	(2,465 ~ 2,521)	(323 ~ 339)	(2,142 ~ 2,182)	
Age, M ± SD	76.8 ± 7.1	79.2 ± 7.9	75.6 ± 6.9	< .001
Sex (Female), n (%)	1,440 (57.1)	261 (77.0)	1,179 (54.3)	< .001
Race/ethnicity, n (%)				< .001
White, non-Hispanic	1,596 (70.1)	171 (50.4)	1,596 (73.1)	
Black, non-Hispanic	548 (21.7)	110 (43.5)	438 (20.1)	
Hispanic	124 (4.9)	21 (10.9)	61 (4.0)	
Other	82 (3.3)	37 (6.2)	87 (2.8)	
Education, n (%)				< .001
Less than high school	558 (22.3)	131 (39.6)	427 (19.7)	
High school	664 (26.5)	87 (26.3)	577 (26.6)	
Some college or vocational school	617 (24.7)	67 (20.2)	550 (25.3)	
College or higher	664 (26.5)	46 (13.9)	618 (28.4)	
Living arrangement, n (%)				< .001
Alone	880 (32.2)	119 (35.2)	689 (31.7)	
With spouse/partner only	1,119 (44.6)	88 (26.0)	1,031 (47.4)	
With others only	335 (13.3)	97 (28.7)	238 (11.0)	
With spouse/partner and with others	250 (9.9)	34 (10.1)	216 (9.9)	
BMI ≥ 30 kg/m^2^, n (%)	713 (28.9)	117 (36.2)	596 (27.8)	0.002
Pain, n (%)	1,261 (50.0)	242 (71.4)	1,019 (46.7)	< .001
Depressive symptoms, n (%)	248 (9.9)	71 (21.2)	177 (8.1)	< .001
Dementia, n (%)	63 (2.5)	37 (10.9)	26 (1.2)	< .001
Hospitalization, n (%)	445 (17.7)	113 (33.4)	332 (15.2)	< .001
Number of ADL impairments, M ± SD	0.3 ± 0.7	1.2 ± 1.3	0.1 ± 0.5	< .001
Number of IADL impairments, M ± SD	0.6 ± 1.2	2.3 ± 1.8	0.3 ± 0.8	< .001
Number of chronic diseases, M ± SD	2.3 ± 1.5	2.9 ± 1.5	2.2 ± 1.4	< .001
Visual impairments, n (%)	159 (6.3)	52 (15.4)	107 (4.9)	< .001
Hearing impairments, n (%)	302 (12.0)	63 (18.8)	239 (11.0)	< .001
Vigorous activity, n (%)	1,047 (41.6)	65 (19.2)	982 (45.0)	< .001

*M* mean, *SD* Standard deviations, *BMI* Body mass index, *ADL* Activities of daily living, *IADL* Instrumental activities of daily living

**Fig. 2:**
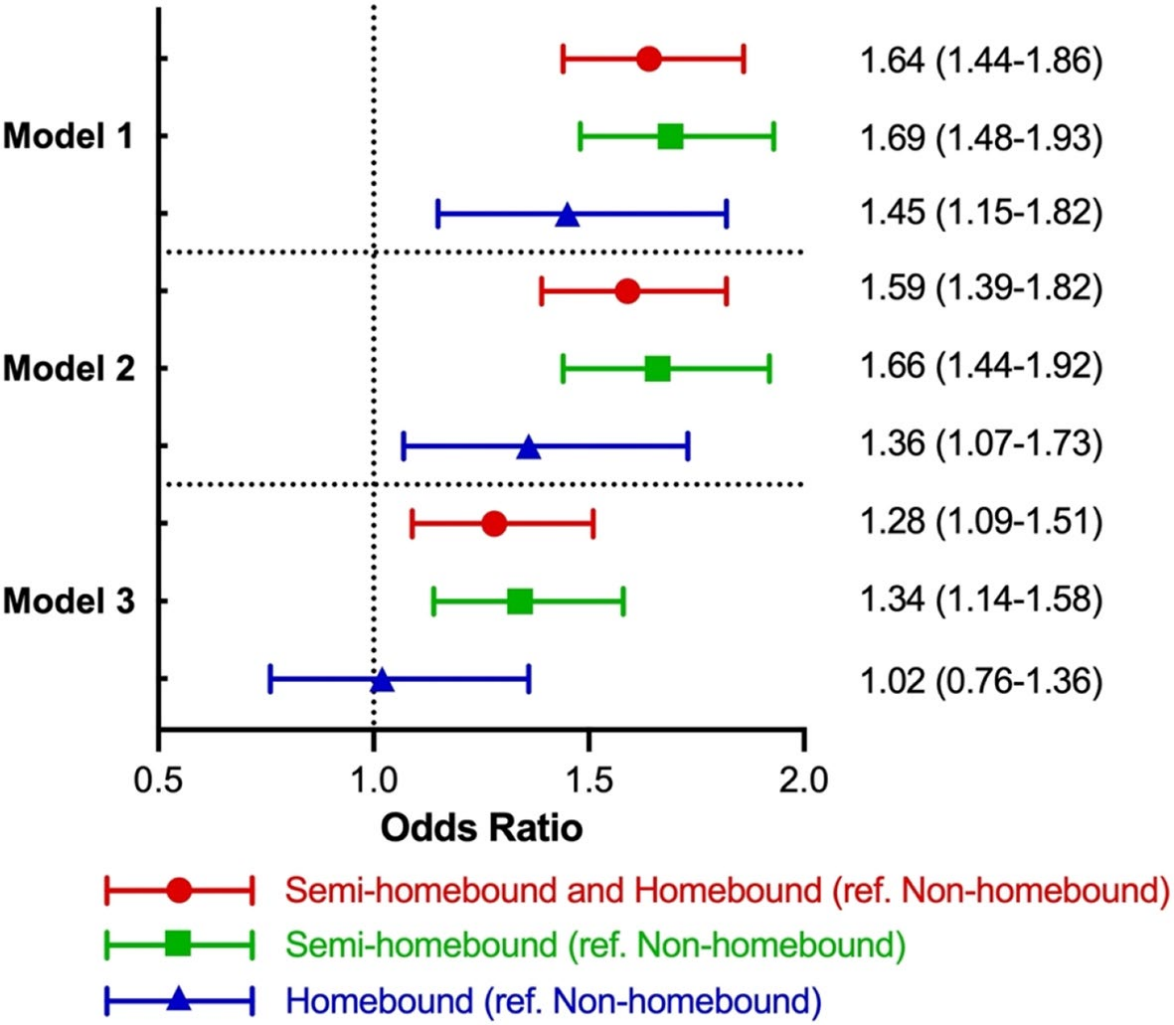
Generalized estimating equation analysis of homebound status predicting falls. The main analysis is represented by the red line with a circle dot. Sensitivity analysis is represented by the green line with a square dot and the blue line with a triangle dot. Model 1: independent variables of interest; Model 2: Model 1 + demographic covariates (age, sex, education, race/ethnicity, living arrangement); Model 3: Model 2 + health-related covariates (BMI, vigorous activity, pain, hospitalization, depressive symptoms, dementia, number of ADL/IADL impairments and chronic illness, hearing and vision impairment)

Figure 2 shows the main and sensitivity analysis results of GEE models using homebound status to predict falls. In the main analysis, prior-year semi-homebound and homebound status significantly predicted the following year's falls in the unadjusted models (OR, 1.64, 95% CI: 1.44–1.86). After adjusting for demographic and health-related covariates, the magnitude of prediction was reduced but remained statistically significant (OR, 1.28, 95% CI: 1.09–1.51). In the sensitivity analysis, the full adjusted model results (Model 3) suggested that compared to non-homebound participants, semi-homebound participants (OR, 1.34, 95% CI: 1.14–1.58) in the prior year had a significantly higher risk of falls in the following year but being homebound was not significantly associated with falls (OR, 1.02, 95% CI: 0.76–1.36).

### Falls as a predictor of semi-homebound and homebound status

A total of 2,916 participants who reported non-homebound at baseline were included in sample 2, and 25% of them had falls. Characteristics of all participants in sample 2 and their comparisons by falls were presented in Table 2 (*N* = 2,916). In sample 2, the mean age of all participants was 75.7 years old. Fifty-six percent were female and 75% were non-Hispanic white. Compared to the participants with no falls, those who experienced falls reported experiencing more pain, depressive symptoms, and hearing problems, and had more ADL/IADL impairment and chronic diseases (*P* < 0.001 for all comparisons).

**Table 2:** Baseline characteristics of participants by falls (Sample 2, *N* = 2,916)

Characteristics	Sample 2			*P* value
	Total	Falls	Non-falls	
	(2,858 ~ 2,916)	(716 ~ 734)	(2,142 ~ 2,182)	
Age, M ± SD	75.7 ± 6.9	75.8 ± 7.0	75.6 ± 6.9	.404
Sex (Female), n (%)	1,628 (55.8)	449 (61.2)	1,179 (54.0)	.001
Race/ethnicity, n (%)				.004
White, non-Hispanic	2,180 (74.8)	584 (79.6)	1,596 (73.1)	
Black, non-Hispanic	543 (18.6)	105 (14.3)	438 (20.1)	
Hispanic	114 (3.9)	27 (3.6)	87 (4.0)	
Other	79 (2.7)	18 (2.5)	61 (2.8)	
Education, n (%)				.705
Less than high school	561 (19.3)	134 (18.4)	427 (19.7)	
High school	768 (26.5)	191 (26.2)	577 (26.6)	
Some college or vocational school	731 (25.2)	181 (24.8)	550 (25.3)	
College or higher	841 (29.0)	223 (30.6)	618 (28.4)	
Living arrangement, n (%)				.017
Alone	938 (32.3)	249 (34.0)	689 (31.7)	
With spouse/partner only	1,359 (46.7)	328 (44.8)	1,031 (47.4)	
With others only	340 (11.7)	102 (13.9)	238 (11.0)	
With spouse/partner and with others	269 (9.3)	53 (7.2)	216 (9.9)	
BMI ≥ 30 kg/m^2^, n (%)	812 (28.4)	216 (30.2)	596 (27.8)	.229
Pain, n (%)	1,469 (50.1)	441 (60.1)	1,019 (46.7)	< .001
Depressive symptoms, n (%)	274 (9.4)	97 (13.3)	177 (8.1)	< .001
Dementia, n (%)	39 (1.3)	13 (1.7)	26 (1.2)	.235
Hospitalization, n (%)	474 (16.3)	142 (19.4)	332 (15.2)	.008
Number of ADL impairments, M ± SD	0.2 ± 0.5	0.3 ± 0.6	0.1 ± 0.5	< .001
Number of IADL impairments, M ± SD	0.4 ± 0.8	0.5 ± 1.0	0.3 ± 0.8	< .001
Number of chronic diseases, M ± SD	2.3 ± 1.4	2.6 ± 1.5	2.2 ± 1.4	< .001
Visual impairments, n (%)	163 (5.6)	56 (7.7)	107 (4.9)	.005
Hearing impairments, n (%)	355 (12.2)	116 (15.9)	239 (11.0)	< .001
Vigorous activity, n (%)	1,303 (44.7)	321 (43.7)	982 (45.0)	.549

*M* Mean, *SD* Standard deviations, *BMI* Body mass index, *ADL* Activities of daily living, *IADL* Instrumental activities of daily living

**Fig. 3:**
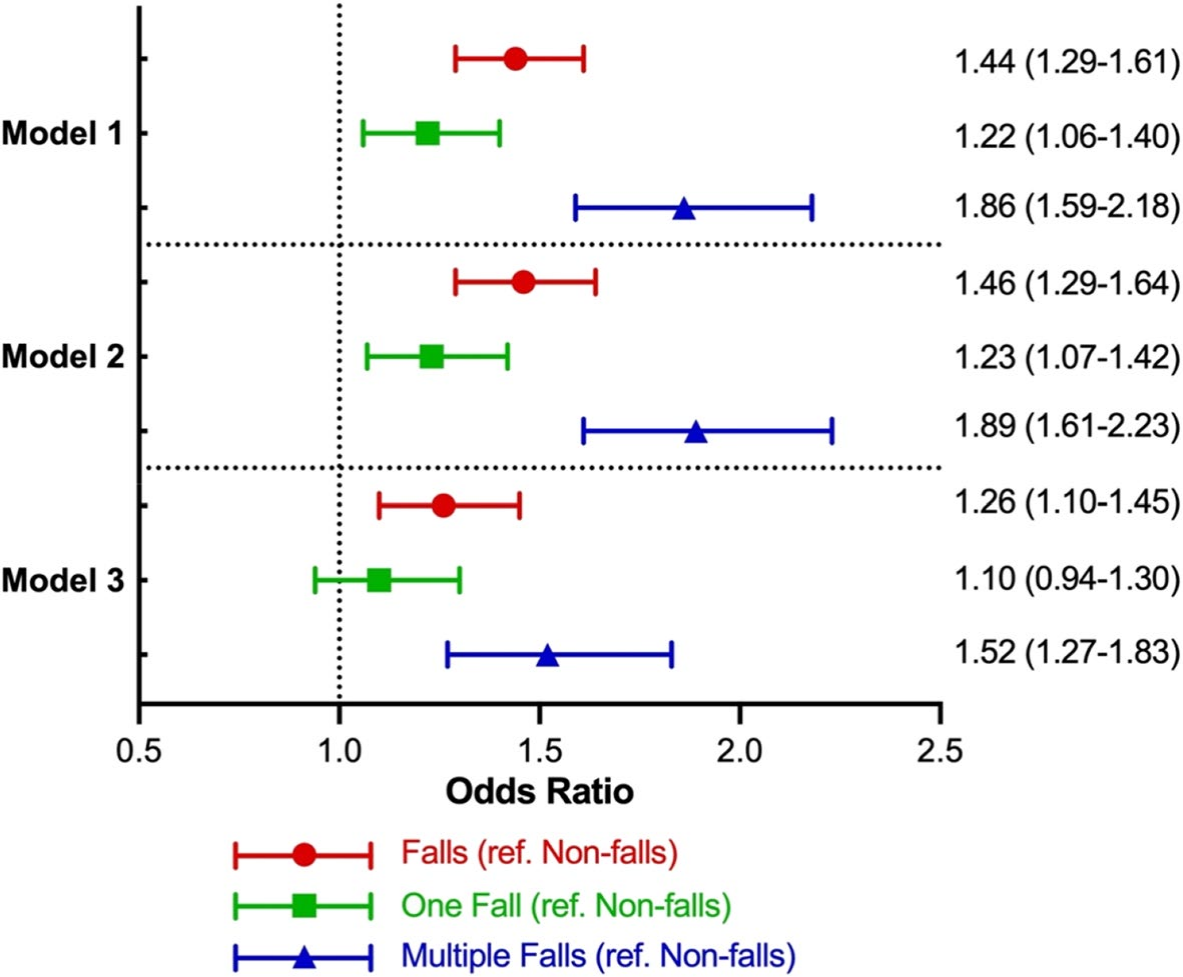
Generalized estimating equation analysis of falls predicting homebound status. The main analysis is represented by the red line with a circle dot. Sensitivity analysis is represented by the green line with a square dot and the blue line with a triangle dot. Model 1: independent variables of interest; Model 2: Model 1 + demographic covariates (age, sex, education, race/ethnicity, living arrangement); Model 3: Model 2 + health-related covariates (BMI, vigorous activity, pain, hospitalization, depressive symptoms, dementia, number of ADL/IADL impairments and chronic illness, hearing and vision impairment)

Figure 3 shows the main and sensitivity analysis results of GEE models using falls to predict semi-homebound and homebound status. In the main analysis, prior year falls significantly predicted the following year’s semi-homebound and homebound status in the unadjusted models (OR, 1.44, 95% CI: 1.29–1.61). After adjusting for demographic and health-related covariates, the magnitude of prediction reduced but remained statistically significant (OR, 1.26, 95% CI: 1.10–1.45). In the sensitivity analysis, compared to the participants who reported no falls in the prior year, those with multiple falls (OR, 1.52, 95% CI: 1.27–1.83) had a significantly higher risk of falls in the following year. However, the prediction effects of falls in the prior year on semi-homebound and homebound status at follow-up did not differ between no falls and one fall.

## Discussion

To our knowledge, this study was the first longitudinal study to determine the bidirectional lagged associations between falls and homebound status using a nationally representative cohort of older adults. Specifically, people with falls in the prior year were at higher risk of developing semi-homebound/homebound status in the following year, and people with semi-homebound/homebound status in the prior year were at higher risk of experiencing falls in the following year. Given that falls and homebound status are preventable, our bidirectional findings shed light on the homebound status as a target of intervention to prevent and reduce falls in later life, and falls as a target of intervention to prevent and reduce the chance of being homebound in the future.

Our findings revealed that homebound status in the prior year was associated with a higher risk of falls in the following year. However, the results from sensitivity analysis suggest that rather than homebound status, semi-homebound status significantly predicted future falls. The semi-homebound population (defined as those who cannot leave home without help) is similar to the homebound population in terms of physical function and disease burdens, such as high mortality and care needs [4]. One possible interpretation for this sensitivity analysis result is that those who are homebound stay indoors, spend a more sedentary lifestyle, and tend to be less active. Therefore, there will be less chance of falls compared to those who are semi-homebound. But those who are semi-homebound tend to be physically vulnerable but still try to go outside and live an active lifestyle compared to those who are completely homebound. Thus, they will have more chances to fall and be at a higher risk of falls overall. Identifying the intensity of a risk factor can improve establish priorities in fall prevention [30], and our findings can provide evidence on tailoring the fall intervention services to semi-homebound older adults. However, the sample size of homebound participants was much smaller than the non-homebound sample (homebound vs. non-homebound: 2.74% vs. 86.55%). It might result in insufficient statistical power to detect the significant associations between homebound status and falls. A study by Zhao and colleagues, although it was a cross-sectional study, supports our findings that both semi-homebound (OR, 1.50, 95% CI: 1.15–1.96) and homebound (OR, 1.67, 95% CI: 1.01–2.75) status were significantly associated with falls in the last month [5]. More studies are needed to examine the inconsistency.

Our findings suggest that falls in the prior year were associated with a higher risk of homebound status in the following year. This finding is consistent with an aforementioned cross-sectional study with a sample of 564 homebound Japanese older adults living with diabetes [11]. Our study used a nationally representative sample, therefore, our results further confirmed the validity of the findings with higher external validity. In addition, our findings showed that older adults with multiple falls had a higher risk of being homebound compared to those who reported fewer falls. This result suggests that those with recurrent falls are vulnerable and may require more healthcare attention [31].

We found that falls were associated with homebound status bidirectionally. Homebound older adults may be limited in their physical capacity [24], and that increases the risk of falls. Falls can lead to injuries, such as hip fractures [3], that cause mobility restrictions. Thus, falls and homebound status may form a vicious cycle and mutually influence each other over time.

This bidirectional relationship between falls and homebound status can be explained by the following mechanisms. Firstly, the homebound status may be the consequence of falling, and vice versa. Falls can cause hip fractures and physical disability [3]. This pattern will directly reduce the mobility capacity and further limit the activities of older adults resulting in homebound status [32]. Although studies of the bidirectional associations between homebound status and falls are lacking, established relationships between falls and disability may explain how this bidirectional relationship works. Homebound status is closely connected to disability [4], which used to be a measurement for homebound status [33, 34]. Previous studies found falls may be associated with disability bidirectionally [3, 35, 36], which further supported our findings. Secondly, falls may be associated with homebound status through shared risk factors, such as balance impairments, disability [26, 37], and depression [15, 38]. Additionally, previous studies found that older adults who are homebound or have fallen tend to have dementia, comorbidity, and depression [4, 39], suggesting these adverse health outcomes may mediate the relationship between falls and homebound status. For example, previous longitudinal studies found that both falls and homebound status were associated with depression bidirectionally [15, 16], suggesting falls and homebound are associated with each other through depression. Our findings also suggested that the association between falls and homebound status may be mediated by some health-related factors (e.g., comorbidity), but the exact mechanism remains to be further determined.

As older adults tend to spend time at home and falls are a serious public health concern [9, 40], our findings have important clinical implications for public health practice and research. First, our finding suggested that older adults may have a reduction in the number of falls within one year if they are no longer homebound. For policymakers, public health workers and homecare providers, it’s important to identify older adults who have difficulty moving independently and provide healthcare service for these older adults to move safely and reduce falls. For example, we can help them to use assistive devices and implement home modifications [34], by removing environmental barriers [32, 41]. Second, future fall intervention studies could further test the effects of reversing homebound status. Previous studies have found that fall intervention programs improved the physical function and quality of life of older adults [42, 43], which shows the possibility of further reducing outdoor mobility problems and social isolation. Third, given the bidirectional relationship between falls and homebound status, preventing falls and homebound status together may have an important clinical implication on healthy aging. Currently, we found that one evidence-based disability prevention program has shown the effect of increasing life-space mobility and falls efficacy simultaneously [44], but we did not find any interventions specifically to improve falls and homebound status simultaneously, which suggests a future research inquiry. We identified some fall prevention programs, especially physical exercise programs. These programs also have shown the potential to increase mobility [45–47], which may help improve homebound status. However, only few mobility programs have tested their effects and efficacy in reducing falls. Therefore, future mobility programs should examine the potential in reducing falls.

This study has several strengths. First, to our knowledge, this study was the first to determine the reciprocal relationship between falls and homebound status using a prospective study design. Second, an adequate and representative sample increased the robustness and generalizability of the study. Third, the study assessed a comprehensive list of covariates, which allowed fairly robust results to be obtained by adjusting for them. However, several limitations should be noted. First, we excluded long-term care facility residents from this analysis because falls and homebound measures were not available for that group. Thus, our results were only generalizable to community-dwelling Medicare beneficiaries in the U.S. [22]. Second, the measurements were self-reported by the participants and may have recall bias and reporting errors. Especially in older adults, a one-year recall time can be too long. They may forget non-fatal falls and underestimate falls status. We recommend collecting falls data using a monthly calendar for future studies.

## Conclusion

The study was the first longitudinal study to determine the bidirectional lagged associations between falls and homebound status using a nationally representative dataset. We found that homebound status is a predictor of future falls and falls can predict future homebound status. Healthcare providers should take fall prevention programs for the homebound older population. Besides, intensive monitoring and tailored interventions for those at high fall risks may help prevent and improve the homebound status.

## Data Availability

The data that support the findings of this study are available from NHATS (https://www.nhats.org/) upon authors’ registration and with the permission of the NHATS research team. The reason for registration is that the NHATS research team wants to document the size and diversity of their user’s community. They also want to contact the user regarding changes to data or documentation or announcements of interest.

## Abbreviations

NHATS: National Health and Aging Trends Study
BMI: Body mass index
ADL: Activity of daily living
IADL: Instrumental activities of daily living
GEE: Generalized estimation equation
M: Mean
SD: Standard deviations
OR: Odds ratio
NA: Not applicable
CI: Confidence interval
